# Behind the scenes of the healthcare COVID-19 pandemic crisis: potential affecting factors of healthcare work sustainability in Romania during 2020–2022

**DOI:** 10.3389/fpsyt.2023.1179803

**Published:** 2023-06-01

**Authors:** Cristina Savu, Iuliana Armaș, Marin Burcea, Daniela Dobre

**Affiliations:** ^1^Faculty of Geography, University of Bucharest, Bucharest, Romania; ^2^Faculty of Administration and Business, University of Bucharest, Bucharest, Romania

**Keywords:** COVID-19, medical staff, cross-sectional survey, panel study, SEM, health perception, work engagement, meaningfulness

## Abstract

**Aim:**

The COVID-19 pandemic represented a great disturbance for medical systems around the world, putting medical personnel on the front lines of the fight against the SARS-Cov2 virus. This fight was particularly impactful in countries with medical systems already facing various challenges, including Romania; where the pandemic unfolded in five waves that severely affected the psychological and physical well-being of medical professionals in terms of overload and continuous exposure to health threats. Against this background, our research aims to identify the mediating role of potential affecting factors of healthcare work sustainability during the change-related uncertainty conditions generated by the COVID 19 crisis. Dynamics and relations of nine carefully selected constructs were tracked along all five pandemic waves in Romania, which span from March 2020 to April 2022. The tested variables and constructs are perception of healthcare workers of their own state of health, their workplace safety, the work–family conflict, the satisfaction of basic needs, the work meaningfulness and work engagement, patient care, pandemic stress and burnout.

**Methods:**

This cross-sectional study is based on an online snowball sampling of 738 health workers from 27 hospitals. Panel research is limited to a maximum of 61 respondents for two successive waves. The analytical part is built on means comparison of analysed variables between all five pandemic waves and an in-depth model to explain the relationships between the variables.

**Results:**

The results indicate statistically significant correlations between the perception of health risks and all selected factors excluding patient care, which seems to be above the own health perception. The factors’ dynamics was followed along all five pandemic waves. The developed model identified that one’s health status satisfaction is a mediator of the family–work conflict and, together, of work engagement. In turn, work engagement plays a significant role in satisfying basic psychological needs and supporting work meaningfulness. Also, work meaningfulness influences the satisfaction of basic psychological needs.

**Discussion:**

Health workers with higher levels of positive perceived health are better at managing pandemic stress, burnout effects and work-family imbalances. Adaptive behaviors and attitudes towards COVID-19 pandemic threats could be identified in later pandemic waves due to the progress in terms of medical protocols and procedures.

## 1. Introduction

The COVID-19 pandemic caused by the SARS-CoV-2 virus appeared in China in December 2019 and spread rapidly throughout the world (1). The disease recorded 525,467,084 confirmed cases and more than 6,285,171 deaths between March 2020 and May 2022 (2), putting humanity in front of an unprecedented health policy crisis (3). The classic sanitary control measures (i.e., social distancing and wearing a gauze face mask), which were introduced with great difficulty by doctors such as Max C. Starkloff aiming to limit the spread of the Spanish flu in 1918 (4), were complemented in modern times by appropriate new treatments and vaccines, which could trigger possible adaptive behaviors and attitudes of medical personnel towards COVID-19 threats in successive pandemic waves.

In Romania, the first case of SARS-CoV-2 infection emerged on February 26, 2020 (5), and by April 2022 the total number of deaths caused by the COVID-19 disease raised to 65,486 (6, 7) (Table 1).

**Table 1:** The highest daily number of COVID-19 cases per wave in Romania (source Ministry of Internal Affairs and Ministry of Health, 2020–2022).

Pandemic waves	Variant of SARS-CoV-2	New daily cases	People hospitalized	People hospitalized in (intensive care units)	Deaths
Wave 1 (March–May 2020)	Alpha	362	–	247	–
Wave 2 (September–December 2020)	Alpha Beta	10,260	12,133	1,130	171
Wave 3 (March–May 2021)	Gamma	6,651	14,165	1,531	237
Wave 4 (September–December 2021)	Delta	18,863	20,962	1902	574
Wave 5 (January–March 2022)	Omicron	40,018	11,884	1,169	215

Being confronted with periodic increases in the number of infected people, hospitals faced both an overload, and the contamination of the medical staff. Moreover, in certain hospitals, the number of medical personnel was reduced due to redistribution to the areas most affected by COVID (e.g., Marius Nasta Hospital, Bucharest) (8). An immediate consequence was the mental and physical overload of the medical staff remaining active in the source hospital (9).

## 2. Research background

Our research is embedded in the behavioral adaptability theoretical background at the individual context-specific level (10, 11).

In the last decades, extensively investigations have been conducted by scholars in the field of adaptability at the individual level (12–18).

Doron (19) defines adaptation as a “dynamic process of change, in order to find balance with the environment and assuming the ability to learn.” In the same way, Gorgos (20) believes that adaptation is an “active, dynamic and creative process, which requires a permanent effort made through the processes of integration and regulation, which makes possible the optimal use of functional reserves, as well as their restoration during the period when the demand ceases.” At the same time, adaptation facilitates the elimination or change of conditions that create problems; perceiving the control of the meaning of the experiences in such a way as to neutralize their problematic character; keeping the emotional consequences of problems within controllable limits (21, 22).

Kiymaz (23) stated that “our brain and body react physiologically and behaviourally to adapt to a social and physical environment that can put your life in danger” ((23), p 1163). In the process of psychosocial adaptation, the individual tends to achieve a harmony between living conditions and internal or external activity. As this harmony is achieved, the degree of adaptability of the individual increases. Psychosocial adaptation also appears as a means of protecting the individual, with the help of which one relaxes and eliminates internal psychic tension, restlessness, destabilizing states (24).

The COVID 19 crisis generated a large amount of change-related uncertainty (25) most dramatically affecting the health professionals put on the front lines of the fight against the SARS-CoV-2 virus. During the COVID 19 pandemic crisis, the medical staff was constantly faced with mighty challenges related to limited resources (26), longer shifts, disturbances in the balance between professional and private life, sleep impairment, and major changes in their working environment (27–29). As changes in the working environment increased dramatically during the COVID 19 crisis, the stress levels also increased. Neuroscience studies ((30), p 384 (31)) show that under increased stress levels, both creativity and the ability to sustain high-level thinking decrease. Stressors have an impact on creative problem-solving skills in difficult situations, so that the ability to multitask is reduced.

Health workers underwent several dramatical changes in their lifestyle, living with the constant fear of contamination, sleep shortening, behavioral changes (32). As a direct consequence, an increase in mental problems has been measured in an abundance of publications investigating the complex ways in which medical personnel were psychologically affected (29, 33). In the context of COVID-19 pandemic, the most frequently recorded consequences were anxiety, stress due to overwork, frustration, discrimination, isolation, lack of contact with family members, pressure and exhaustion due to high risk of infection and inadequate protection against contamination, post-traumatic stress disorder, psychological distress and depression, sleep disorders, and fear (28, 34–49).

According to other studies, during COVID-19, the possibility of infecting family and friends traumatized medical workers (50) and diminished the level of their psychological well-being that contributes to safety, happiness, satisfaction and increased work performance (51).

Therefore, the burnout increased by 25–30% compared to the period before the pandemic (52, 53), and the most prominent sources of burnout were the cumulative work tasks, uncertainties caused by the pandemic, work-family imbalance and strained relationships at work (54). Taylor (49) mentioned, referring to a study on natural disasters and making a correlation with the COVID 19 pandemic crisis, that 10% of people who have gone through traumatic events, in the context of increased emotional stress and social problems, can immediately or later develop severe psychological problems, such as post-traumatic stress disorders, anxiety disorders, restlessness, sleep disorders, mood disorders.

On the other hand, support, job satisfaction, and an improvement in the self-esteem of medical personnel, were listed as protective factors against burnout (55), showing that the pandemic had serious implications for patient care and job satisfaction (56). The association between the doctor’s burnout scale with work involvement and the quality of care given to patients is important for highlighting the general efficiency of medical service providers (57–59).

Designed against this background of pandemic induced uncertainty, our study aims to identify potential affecting factors in physicians’ work sustainability during the COVID-19 pandemic in Romania in terms of health workers’ perception-related variables (their own state of health), perceived threat of COVID-19 (pandemic stress), work–family conflict, patient care, work engagement, meaning of and commitment to work, satisfaction of basic psychological needs, as well as psychological and professional burnout of healthcare professionals during the COVID-19 pandemic, at the level of 27 hospitals in Romania.

An important attempt is to identify possible relationships between the nine tested constructs and adaptive behaviors and attitudes towards COVID-19 pandemic threats due to the progress of medical protocols, from the way the tested factors fluctuated during the 5 pandemic waves, both at the level of the independent samples and among the subjects in the panel. An in-depth structural model was built to explain the relationships between the selected constructs.

## 3. Methodology

### 3.1. General approach

This study undertakes a cross-sectional approach, applying repeated surveys with different respondents in each wave on a total of 738 subjects. This is complemented by a panel-oriented approach (participants who responded in two successive pandemic waves), totalling a maximum of 61 respondents per successive waves.

Participants were recruited from the medical personnel of 27 hospitals in Romania. These professionals were involved in the fight against the pandemic along the five waves, beginning in May 2020 and ending in April 2022. We applied the on-probability snowball sampling technique (60), sharing the questionnaire online for voluntary participation. Respondents were asked to read and agree to informed consent and the statement regarding the processing of the data collected through the survey. Upon request, additional information was made available for respondents *via* email.

### 3.2. Measures

#### 3.2.1. Risk perception

The questionnaire investigated the perception of various risks to which participants are exposed, the work–family conflict, the satisfaction of basic needs, the work meaningfulness and work engagement, patient care, pandemic stress and burnout. This section describes the operationalisation of these aspects. The measuring of *risk perception* focused on the level of perceived state of health, perceived safety versus workplace insecurity, the perceived level of danger, and the existence of an “unhealthy” environment (e.g., with risk of injury, death, health damage) during the SARS-CoV-2 pandemic. An example of an item is: “Describe your workplace in relation to the crisis generated by the Coronavirus pandemic: Dangerous, Safe, Risky, Unhealthy, Uncertain, Risk of death.” Answers were scored on a 5-point Likert scale, from 1 (strongly disagree) to 5 (strongly agree).

#### 3.2.2. Work–family conflict

Work–family conflict was measured with the Work Family Conflict Scale proposed by Carlson and Kacmar (61). The scale is based on six dimensions of conflict, resulting from the combination of three forms of work–family conflict (time, strain, and behavior), and two directions of work–family conflict (work-family interference and family-work interference). In the current study, only 6 items of the Work–Family Conflict Scale were used, considering only 2 dimensions of the scale: time based on work-family interference, and strain based on work-family interference. The current study aims to highlight the impact of the activities carried out by medical personnel at work on their family, as well as the state of tension and stress resulting from this interaction (e.g., “Work keeps me away from my family for too much time,” “The time I spend at work does not allow me to participate enough in family activities,” “When I get home from work, I am too tired to participate in family activities,” “It happens that the stress at work also affects me at home, so that I can no longer do what I like or what I enjoy).” All items are scored directly and involved responses on a five-point Likert scale, from 1 (never) to 5 (always).

#### 3.2.3. Work engagement

Work engagement was measured with the short version of the Utrecht Work Engagement Scale (UWES-9) (62, 63), which refers to three factors of work engagement, with three items each: vigor (e.g., “When I wake up in the morning, I feel like going to work.”), dedication (e.g.: “I am proud of the work I do”), and absorption (e.g., “I am fully involved when I work”). All the items are scored directly, on a 7-point Likert scale, from 0 (never) to 6 (always).

#### 3.2.4. Basic psychological needs satisfaction

Basic needs satisfaction was measured using the Basic Psychological Needs Satisfaction Scale and the Frustration Scale (64). The three subscales focus on autonomy (e.g., “At work, I feel a sense of choice and freedom in the things I undertake”), competence (e.g., “I feel confident that I can do things well at work”), and relatedness/referring to the extent to which a person feels connected with and valued by others (e.g., “I feel that people who care at work care about me too”). Each of the three subscales was assessed *via* satisfaction questionnaires with 4 directly scored items on a seven-point Likert scale from 1 (strongly disagree) to 7 (strongly agree). Thus, participants report the extent to which their three basic needs were met in the last weeks at work.

#### 3.2.5. Work meaningfulness

Work meaningfulness was measured with the Work inventory and the Meaningful work scale (65), which introduces three components of meaning at work: positive meaning (e.g., “I have a good sense of what makes my work meaningful”), gaining meaning through work (e.g., “My work helps me understand myself better”) and better motivations (e.g., “I know my work makes a positive difference in the world.”). The directly scored items required answers on a five-point Likert scale, from 1 (absolutely not true) to 5 (absolutely true).

#### 3.2.6. Patient care

Patient care was measured with the Patient Care scale (66), an 8-item scale that investigates suboptimal patient care practices (five items, e.g., “We did not fully discuss treatment options.”) and patient care attitudes (three items, e.g., “We paid little attention to the social or personal aspects of the illness impact.”). All the items were scored directly, on a 5-point Likert scale, from 1 (never) to 5 (weekly).

#### 3.2.7. Pandemic stress

Pandemic stress was measured with the corresponding Stanford Acute Stress Reaction Questionnaire – SASRQ, which was developed and validated by Cardena et al. (67) to assess psychological symptoms experienced following a traumatic episode. The SASRQ instrument investigates dissociation (e.g., subjective feeling of numbness, detachment and lack of emotional responsiveness, reduced awareness of the environment, derealization, depersonalization, and dissociative amnesia - 10 items), reexperiencing trauma (6 items), avoidance (6 items), hyper anxiety (6 items), anxiety and impairment of functioning (2 items). The questionnaire was initially applied in the context of natural disasters (floods), but we adapted it to fit the context of traumatic episodes related to the COVID-19 pandemic crisis. All the items are directly scored, on a 6-point Likert scale, from 0 (“I have not experienced/experienced the respective condition.”) to 5 (“I have experienced the respective condition very often.”). This scale was introduced in the research design starting with the second pandemic wave, to measure if new waves lead to an increase in the stress felt by medical professionals.

#### 3.2.8. Burnout

The Burnout Scale [22-item *MBI - Maslach Burnout Inventory* (68)] was originally developed to measure burnout as a specific type of response to occupational stress among human service professionals. We used the 9-item short version of the Maslach Burnout Measurement Inventory (69, 70), with three subscales (exhaustion, cynicism, and inadequacy, including feelings of overwhelming emotional exhaustion, feelings of cynicism and detachment from the workplace defined as depersonalization, and a sense of ineffectiveness and reduced personal fulfilment). Each subscale has 3 directly scored items, measured on a 7-point Likert scale, from 0 (never) to 6 (daily). This scale was introduced in the research design starting with the third pandemic wave, to test whether the persistence of the pandemic waves leads to various degrees of professional exhaustion.

We tested the reliability of the structure by assessing the internal consistency of all applied scales using Cronbach’s alpha. The Cronbach’s alpha of each construct was higher than the recommended level of 0.7.

### 3.3. Data collection

**Figure 1:**
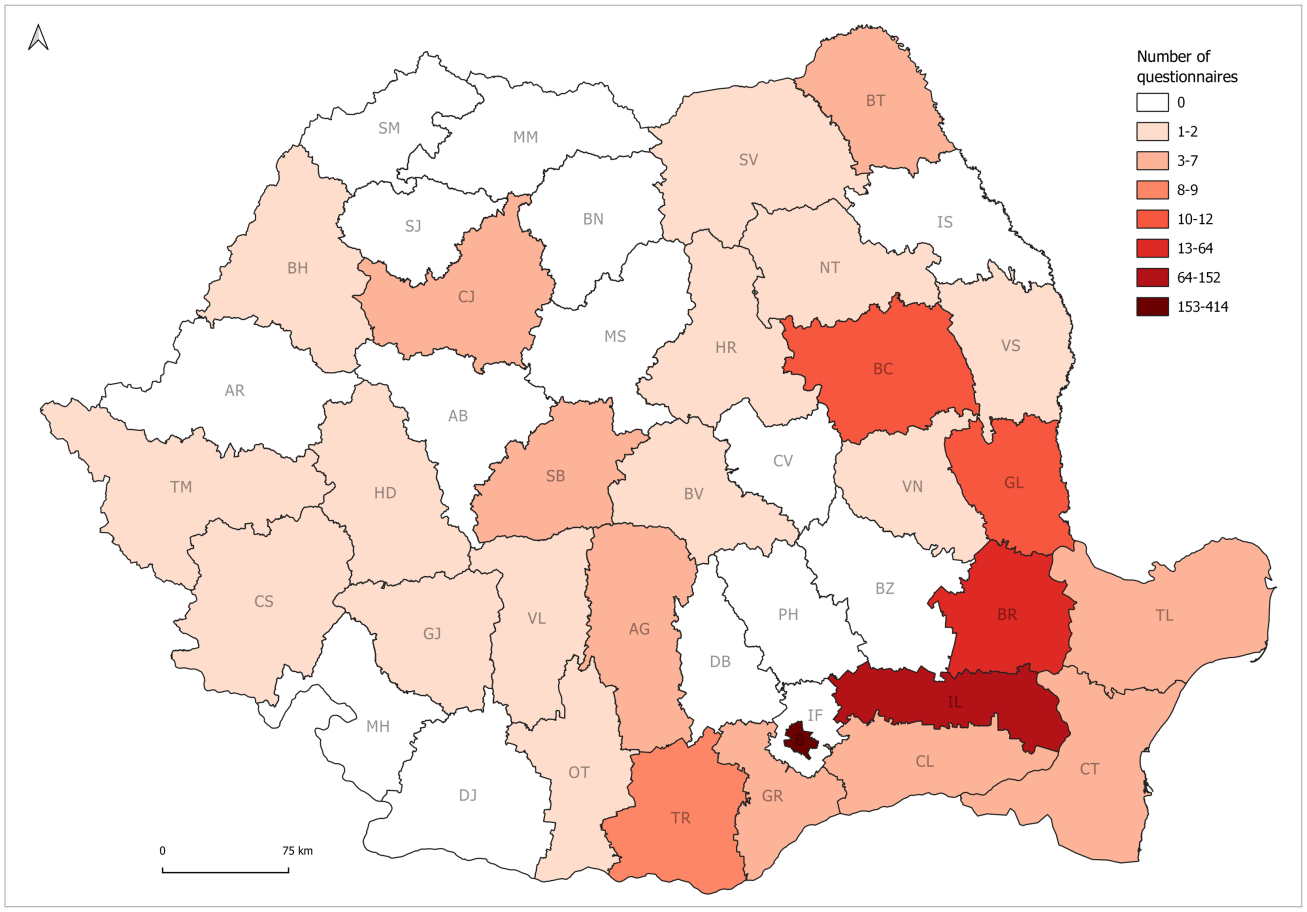
Geographical distribution of respondents.

The data was gathered online during the five pandemic waves using Google Forms for each of the corresponding five rounds, and mainly came from Bucharest (425) and the neighbouring counties: Ialomița (152), followed by Brăila (77), Galați (10), but also from other counties such as Bacău (6), Teleorman (6), Constanța (5), Tulcea (5), Sibiu (5), Călărași (5), Giurgiu (4), Argeș (3), Bihor (2), Neamț (2), Vaslui (2), Olt (2), Brașov (2), Timiș (2) (Figure 1).

The first survey took place between March and May 2020, with a total of 216 healthcare workers. The second round of data collection unfolded in September 2020–January 2021, with 121 respondents, of which 18 subjects were on the panel (i.e., those who took part in the survey in two successive pandemic waves, namely waves 1 and 2).

The third survey took place from February to May 2021, focusing on 195 respondents, with only 6 participants in the panel between waves 2 and 3 (i.e.,9.37% of wave 2, and 3.09% of the third wave).

The fourth round of data collection took place between September and December 2021, and 68 healthcare workers completed the survey, of whom 6 were included in the corresponding panel (i.e., those who repeated in waves 3 and 4). They accounted for 3.09% respondents from the third wave, and 8.82% of respondents from the fourth wave.

The final stage of data collection unfolded between January and March 2022: 138 healthcare workers completed the survey and 31 were panel respondents (i.e., subjects who repeated in waves 4 and 5, respectively 45.58% of respondents from the fourth wave, and 22.46% from the fifth wave). At large, the number of people who participated in the survey in at least two successive pandemic waves (panel participants) reached 61 respondents (respectively 8.26%).

### 3.4. Analytics

Analytical procedures were computed using the IBM^®^ SPSS^®^ Statistics and Jamovi v1.6.23 softwares (71). The computation procedures focused on absolute and relative frequency, means, standard deviations, and normality indicators (i.e., skewness and kurtosis). We calculated the bivariate Pearson correlations (*r*) across the study variables, following Cohen’s (72) benchmarks for interpretation: weak correlation (*r* < 0.3), moderate correlation (0.3 < *r* < 0.55), strong correlations (*r* > 0.5).

The analyses relied on two non-parametric statistical hypothesis tests. The Mann–Whitney U test was applied to explore perception differences at the level of cross-sectional samples during the five pandemic waves. The Wilcoxon signed rank test was applied for the comparative analysis of health status perception of medical staff participating in two consecutive waves.

Using nonexperimental data, causal relationships were examined with path analysis using the Jamovi open software. We developed a model of hypothesized causes in order to test the coping role during the COVID-19 pandemic of health workers’ perception regarding their own state of health in relation with work engagement, family-work relations, basic psychological needs satisfaction, and work meaningfulness. The constructs and their indicators were specified and estimated, followed by structural relationships in the model, which were obtained using the same steps. Further on, the hypothesized model of relations was statistically tested to determine the extent to which it was consistent with the data. We first applied the path analysis to test the initial hypothesized model. Next, the latent variable structural equation modelling - SEM was applied to accurately identify the relationships in the system.

The SEM comprises both a measurement model and a structural model. It was used to analyse the relationships between observed variables (derived directly from measurements) and latent variables (constructs that can be measured indirectly by determining their influence on the responses of the measured variables) (73–77). The applied methodological steps were: (1) model identification, (2) parameter estimation, (3) model-fitting, (4) model redefinition, and (5) interpretation of results. In the case of SEM, we followed the five step-model specification, firstly defining the independent and dependent variables; and continuing with the same next steps. We used standardized coefficients to increase comparability and to make inferences regarding the strength of identified relationships (i.e., variables with mean = 0 and standard deviation = 1). The fit of the model to the field data was measured using the chi-square test (78), the comparative Tucker-Lewis Index [TLI; (79)], the comparative fit index [CFI; (80, 81)], the root mean square error of approximation related to the residual in the model [RMSEA; (82)], and the standardized root mean squared residual (SRMR; 75, 76, 83).

## 4. Results

### 4.1. Sample

The 738 completed questionnaires that resulted from the data collection phase showed that the sample is dominated by women (74.5% respondents, mirroring the predominantly female structure of the health personnel in Romania, with 70.5% women among doctors in 2020). Participants with ages of 40–49 years totalled 42.68% of the sample. Most of the medical staff graduated from post-secondary schools (46.61%), and only a third from college (29.40%). The other study levels were represented as follows: postgraduate studies (14.76%), secondary education (7.99). These numbers also indicate the age structure of specific occupations in the medical sector: respondents aged up to 20–29 years mainly have secondary education (post-secondary school); 10.70%, having the lowest average period of employment 0–1 year (20–29 years) (Table 2).

**Table 2:** Frequency distribution of socio-demographic characteristics on research waves.

Variables		Total (738)	Wave 1 (216)	Wave 2 (121)	Wave 3 (195)	Wave 4 (68)	Wave 5 (138)
Gender	M	25.61	30.60	29.00	24.10	25.00	21.74
	F	74.39	69.40	92.00	75.90	75.00	78.26
Education	Secondary education	7.99	15.30	8.20	3.60	2.94	5.07
	Post-secondary studies	46.61	42.60	43.00	62.10	48.52	39.85
	University studies	29.40	28.70	31.40	25.10	33.82	32.60
	Postgraduate studies	14.76	13.40	17.40	9.20	14.70	22.46
Position	Caregiver, nurse, stretcher bearer, registrar	21.54	9.40	9.10	8.10	23.53	28.26
	Medical assistance, paramedic	73.57	71.76	67.76	74.36	75	63.04
	Doctor	5.82	19.00	23.20	5.60	1.47	2.90
Age	20–29 years old	10.70	10.70	9.10	7.10	8.82	18.11
	30–39 years old	26.83	30.10	45.50	15.10	17.64	26.81
	40–49 years old	42.68	44.10	37.30	49.40	51.47	32.20
	50–59 years old	18.43	14.40	8.30	27.80	17.64	21.74
	>60 years old	1.35	1.00	–	1.00	2.94	1.44
Professional experience	0–1 years	5.01	4.20	2.50	5.60	2.94	8.69
	1–3 years	10.56	9.30	18.20	8.70	5.88	10.87
	3–5 years	17.47	14.81	25.60	16.49	13.23	18.11
	>10 years	66.93	71.80	53.70	69.20	77.94	62.32

Respondents aged 40–49 years have the highest share (24.12%) in the category of post-secondary school. The lowest weight of those with secondary school education (2.43%) is in the age category 50–59 years. Results underline that the positions that do not require specialized studies (unqualified) in the medical system are occupied by young people, who are in the early stages of employment, look out for new job opportunities and are less likely to fill their current position for long periods. The best represented professional categories in this study were nurses (68.29%) and doctors (18.56%), the rest of 13.13% being paramedics, stretcher bearers, and registrars. However, these percentages change from one pandemic wave to another.

Medical doctors represented only 1.47% of the participants who completed the survey during the fourth wave, they were best represented in the second wave (23.20%) (Table 2).

Regarding seniority at work, the most numerous were people with seniority >10 years, followed by seniority of 3–5 years, most of them being medical assistants, followed by doctors.

### 4.2. Descriptive statistics

Overall, the distribution of answers by pandemic waves is relatively balanced, but there are also particularities that need to be considered: medical assistants provided the most answers in the 4th pandemic wave (75%), and doctors in the 2nd wave (23.20%).

The descriptive analysis (Table 3) shows that the highest values in terms of the mean of work - family conflict scale were recorded during the 2nd and 4th pandemic waves, while the means regarding the satisfaction of basic psychological needs, the perception of the personal state of health, meaning of and commitment to work have higher values in waves 1 and 3. Workplace engagement and the perception of the workplace achieved higher values in waves 2 and 3. Patient care has higher values in the first two waves, and professional burnout has higher values towards the end of the pandemic, during waves 4 and 5. On the other hand, stress associated with COVID surged during the middle of the pandemic, in waves 2 and 4.

**Table 3:** Descriptive analysis on research waves.

Scale (no respondents)	Total mean (SD)	Wave 1 mean (SD) 216	Wave 2 mean (SD) 121	Wave 3 mean (SD)195	Wave 4 mean (SD)68	Wave 5 mean (SD) 138
Perception of the personal state of health (738)	7.57 (1.69)	8.07(1.28)	7.09 (1.93)	7.64 (1.44)	6.75 (2.08)	6.97 (1.88)
The perception of the workplace (738)	34.67 (7.12)	30.7 (6.92)	33.0 (6.42)	32.9 (6.30)	33.3 (5.79)	30.5 (6.61)
Work–family conflict scale (738)	21.67 (6.09)	19.8 (6.47)	22.2 (5.69)	22.5 (5.72)	22.9 (5.78)	22.4 (5.96)
Workplace engagement (738)	42.26 (8.60)	4.36 (8.52)	41.8 (8.24)	43.1 (8.28)	40.0 (8.33)	40.5 (9.18)
Scale of satisfaction of basic psychological needs (738)	66.78 (12.72)	68.7 (12.7)	65.9 (13.5)	67.5 (11.4)	65.1 (12.5)	64.3 (13.5)
The work and meaningful inventory (738)	42.01 (5.79)	42.4 (5.66)	41.9 (6.24)	42.4 (5.45)	41.3 (5.64)	41.3 (6.13)
Patient care (591)	21.11 (5.55)	19.8 (8.71)	20.4 (8.89)	18.5 (9.09)	17.0 (9.74)	10.7 (6.04)
Stanford acute stress reaction questionnaire (555)	53.67 (36.94)	7.0 (22.1)	55.4 (38.2)	48.2 (33.5)	62.5 (39.1)	57.5 (38.1)
Maslach burnout inventory (415)	21.28 (12.92)	–	2.18 (6.68)	19.1 (12.6)	24.2 (12.9)	23.2 (13.2)

### 4.3. Inferential results

Inferential analysis tested the importance of one’s own state of health in relation to all selected factors and the dynamics of these variables during the five pandemic waves, both at the level of independent samples and in the panel study, to identify possible adaptive behaviors and attitudes towards the COVID-19 pandemic threats. The path analysis and the latent variable structural equation modelling - SEM were applied to explain the relationships between the selected constructs.

#### 4.3.1. Health perception

The fundamental hypothesis is that, in the context of the COVID-19 pandemic, perception of one’s own state of health is pivotal for the perception of all other considered variables: the perception of the specific risks at work, the felt state of pandemic stress, satisfaction of basic psychological needs, meaning of and commitment to work, patient care, but also of the conflict between work and the family life.

This set of working hypotheses are supported by significant direct and indirect proportional correlations (with various degrees of strength) between the perception of one’s state of health and almost all other variables, with one exception (Table 4). Patient care seems to be above the own health perception, but it is significantly, moderately negatively correlated with stress reaction, and significantly, weakly negatively correlated with burnout (Table 4).

**Table 4:** Correlation Pearson.

Correlations		How satisfied are you with your state of health?	The perception of the work place	Work–family conflict scale	Workplace engagement	Scale of satisfaction of basic psychological needs	The work and meaning inventory	Patient care	Stanford acute stress reaction questionnaire	Maslach burnout inventory
How satis fied are you with your state of health?	Pearson Correlation	1	−0.163^**^	−0.283^**^	0.298^**^	0.271^**^	0.196^**^		−0.328^**^	−0.265^**^
	Sig. (2-tailed)		0.000	0.000	0.000	0.000	0.000	0.117	0.000	0.000
	N		525	525	525	525	525	416	384	304
The perception of the workplace	Pearson Correlation		1	0.501^**^	−0.155^**^	−0.139^**^	−0.004	0.107^*^	0.314^**^	0.294^**^
	Sig. (2-tailed)			0.000	0.000	0.001	0.921	0.03	0.000	0.000
	N			525	525	525	525	416	384	304
Work–family conflict scale	Pearson Correlation			1	−0.265^**^	−0.239^**^	−0.005	−0.054	0.370^**^	0.423^**^
	Sig. (2-tailed)				0.000	0.000	0.250	0.274	0.000	0.000
	N				525	525	525	416	384	304
Workplace engagement	Pearson Correlation				1	0.690^**^	0.643^**^	0.086	−0.281^**^	−0.453^**^
	Sig. (2-tailed)					0.000	0.000	0.080	0.000	0.000
	N					525	525	416	384	304
Scale of satisfaction of basic psychological needs	Pearson Correlation					1	0.610^**^	0.108^*^	−0.258^**^	−0.299^**^
	Sig. (2-tailed)						0.000	0.028	0.000	0.000
	N						525	416	384	304
The work and meaning inventory	Pearson Correlation						1	0.058	−0.168^**^	−0.335^**^
	Sig. (2-tailed)							0.238	0.001	0.000
	N							416	384	304
Patient care	Pearson Correlation							1	−0.022	−0.105
	Sig. (2-tailed)								0.694	0.105
	N								309	241
Stan ford acute stress reaction questionnaire	Pearson Correlation								1	0.559^**^
	Sig. (2-tailed)									0.000
	N									304
Maslach burnout inventory	Pearson Correlation									1
	Sig. (2-tailed)									0.000
	*N*									304

Health workers with higher levels of positive perceived health are better at managing pandemic stress (−0.328; *p* = 0.001) and burnout effects (−0.265; *p* = 0.001). In the same acceptance, a positive health perception balances family-work conflicts (−0.283; *p* = 0.001) and negative workplace engagements (−0.163; *p* = 0.001). Work engagement is also negatively correlated with burnout (−0.453; *p* = 0.001) and pandemic stress (−0.282; *p* = 0.001).

Perceived health and work engagement are positively correlated with the satisfaction of basic psychological needs (0.271, *p* = 0.001 and 0.690; *p* = 0.001, Table 4). The perception of workplace highly positively correlates with the work versus family conflict, drawing attention to the fact that the workplace fulfils the function of a second family (0.501; 0.0001). Intuitively, the meaning of work negatively correlates with the effects of burnout (−0.335; *p* = 0.001).

These results are best expressed among doctors, who display high levels of meaningful work and work engagement. Such positive attitudes towards work are shown by the following percentages: 50.73% of responders are highly involved in their work, 36.02% being excited when they work, 30.88% state that they want to go to work when they wake up in the morning, 28.67% are happy when they work intensively, and 11.76% of the doctors answered that they are full of energy at work.

In terms of work significance, 59.55% of the medical personnel declared that they have a career full of significance, 57.35% of doctors stated that their work has a positive impact in the world. Also 50.73% know the significance of their work and 48.52% recognize the contribution of their work to the meaning of life.

#### 4.3.2. Dynamics of health perception

An important task of our research was to capture the dynamics of health perception (“How satisfied are you with your state of health?”) during the 5 pandemic waves, both at the level of independent samples (based on the Mann–Whitney U test) and in the panel study (*via* the Wilcoxon signed rank test).

Results summarized in Table 5 (the value of p associated with the Mann–Whitney U test is listed) show statistically significant differences in terms of health assessment scores between the first wave and the other four waves, and between wave 3 and the next two ones, as described below.

**Table 5:** Perception of one’s own health state.

A. Different pandemic waves						B. Different consecutive waves (panel samples)					
A.	Wave 1	Wave 2	Wave 3	Wave 4	Wave 5	B.	Wave 1	Wave 2	Wave 3	Wave 4	Wave 5
Wave 1		*0.000*	*0.000*	*0.000*	0.183	Wave 1		*0.039*	0.378	*0.009*	NA
Wave 2			0.506	0.092	*0.027*	Wave 2			1,000	0.402	NA
Wave 3				*0.024*	*0.027*	Wave 3				0.138	NA
Wave 4					0.438	Wave 4					NA
Wave 5						Wave 5					

(A) Different pandemic waves; (B) different consecutive waves (panel samples used the Wilcoxon signed rank test, *p* < 0.05). *NA, no answer. The italic values are statistically significant (*p*<0.05).

Certain adaptive behaviors and attitudes towards the COVID-19 pandemic threats are visible during waves 2, 4 and 5 (Mann–Whitney U test *p* < 0.05). Respondents acknowledge that they have coped better with latter pandemic waves due to the progress in terms of medical protocols and procedures. At the beginning of the pandemic there was some lack of confidence and a high degree of scepticism regarding the perception of one’s own health state. Also, waves 1 and 3 are significantly different from waves 4 and 5 (Table 5A). A decrease in the satisfaction level regarding the state of health can be observed during the last two pandemic waves, compared to the onset of the COVID-19 pandemic (wave 1). However, it should be highlighted that the maximum values of health state declared in the self-assessment at the beginning of the pandemic may be the result of a cognitive dissonance effect, which is a psychosocial phenomenon of denying a possible personal vulnerability (84). The perception of one’s own state of health fluctuates from wave to wave, maintaining lower values than in the initial state. Thus, although the medical personnel was confronted with a shortage of knowledge on adequate mitigation procedures and treatment protocols during the first pandemic wave, they displayed a compensatory overconfidence in their own state of health (85), which may have helped the fight against the virus. The lower values related to one’s own health state recorded during the 2nd wave indicate a delay in these concerns, which increased in the next wave, on the grounds that experience gained in previous waves makes us more confident in preventing disease. Lower health state-related values correspond to waves 4 and 5, suggesting an adaptation to the situation, reflected by a decrease in the concern for one’s own health.

The results of the Wilcoxon signed rank test, show that statistically significant differences were recorded between the answers collected in the panel during waves 1, 2 and 4 (Table 5B). These findings are explained by the fact that people were more scared at the very beginning of the pandemic, having the feeling that their health would be seriously affected. In the fourth wave, they already adapted to the pandemic conditions, believing that their health will not be severely impacted by the SARS CoV-2 virus.

The perception of the state of health and the perceived danger at work during the pandemic was tested using the Mann–Whitney U test. The results show that there are significant differences (*p* < 0.05) in the scores obtained for these variables between the following waves: wave 1 and waves 2, 3; wave 2 and wave 5; wave 3 and wave 5 (Table 6A).

**Table 6:** Perception of workplace safety.

A. Different pandemic waves						B. Different consecutive waves (panel samples)					
A.	Wave 1	Wave 2	Wave 3	Wave 4	Wave 5	B.	Wave 1	Wave 2	Wave 3	Wave 4	Wave 5
Wave 1		*0.015*	*0.002*	0.058	0.970	Wave 1		0.084	0.529	*0.018*	0.313
Wave 2			0.970	0.925	*0.024*	Wave 2			0.262	0.937	0.698
Wave 3				0.829	*0.005*	Wave 3				0.919	0.250
Wave 4					0.060	Wave 4					0.499
Wave 5						Wave 5					

(A) Different pandemic waves; (B) different consecutive waves (panel samples used the Wilcoxon signed rank test, *p* < 0.05). The italic values are statistically significant (*p*<0.05).

The mean values show that the perception of workplace safety against the effects of the pandemic in waves 1 and 5 was lower than in waves 2, 3, 4. The transition from the COVID-19 Alpha variants specific to the first wave, to the Omicron variant characteristic to the last wave had a significant impact on health workers. Nonetheless, at the beginning of the pandemic, COVID-19 was something new and quite dangerous, causing the medical staff to doubt the safety of their workplace. The measures mandated to combat the pandemic were also very strict during the first wave (lockdown), whereas the Omicron variant of the virus, which emerged at the end of the pandemic, was perceived more as an easy flu, meaning that the perception of the danger to one’s health at work decreased. In waves 2, 3, and 4, the COVID-19 isolation measures were no longer very strict in Romania, which led to higher levels of health-related self-assessment.

The Wilcoxon signed rank test recorded statistically significant differences between responses regarding the workplace perception of people in the panel between wave 1 and 4 (Table 6B), in the sense that the perception of dangerousness at work decreases in wave 4 compared to wave 1. These findings could be related to the new, less aggressive variants of SARS-CoV-2, but also to the advancement of knowledge, increased treatment capacities, and more effective measures to mitigate the pandemic (85, 86). If we compare waves 1 and 4 to waves 2, 3 and 5, we can identify a sharpening of perception regarding the state of health, but also concerns about “how risky is the workplace in the context of the pandemic” and “whenever unforeseen events can occur.” Also, between wave 1 and wave 4, it appears that the respondents in the panel sharpen their perception of job security, which may come as a result of an adaptation processes.

#### 4.3.3. Work–family conflict, work engagement and significance, psychological needs, patient care, pandemic stress and burnout dynamics

Another research aim was to measure the dynamics of conflict between professional and personal life, work engagement and commitment, work significance, satisfying basic psychological needs, patient care, pandemic stress and burnout during the 5 pandemic waves, both at the level of independent samples (based on the Mann–Whitney U test) and in the panel study (*via* the Wilcoxon signed rank test).

The dynamics of the conflict between work and personal life during different pandemic waves revealed statistically significant differences (*p* < 0.05) between wave 1 and waves 2, 3 and 5 (Table 7A). The balance between work and family was seriously affected during the first pandemic wave, a fact that can be explained by the involvement of all available resources in the battle against an unknown impactful virus. The conflict considerably diminished in the fifth wave, and as a result of the “habit” effect. Starting with the 2nd and 3rd waves, the pandemic limitation measures relaxed (87), increasing the contamination risk of the population and causing additional pressure on the medical system. As an immediate consequence, health workers were affected, and their work conditions were subject to various risks. This explains the statistically significant differences recorded between these waves and the last one, during which the Omicron variant no longer raised major problems for the health system.

**Table 7:** The work versus family conflict scale.

A. Different pandemic waves						B. Different consecutive waves (panel samples)					
A.	Wave 1	Wave 2	Wave 3	Wave 4	Wave 5	B.	Wave 1	Wave 2	Wave 3	Wave 4	Wave 5
Wave 1		*0.015*	*0.002*	0.058	*0.000*	Wave 1		0.569	0.889	0.352	0.522
Wave 2			0.970	0.925	*0.024*	Wave 2			0.107	0.724	0.844
Wave 3				0.829	*0.005*	Wave 3				0.755	0.625
Wave 4					0.060	Wave 4					0.213
Wave 5						Wave 5					

(A) Different pandemic waves; (B) different consecutive waves (panel samples-Wilcoxon signed rank test, value of *p* < 0.05). The italic values are statistically significant (*p*<0.05).

The results of the Wilcoxon signed rank test suggest that there are no statistically significant differences regarding the conflict between family life and the time spent at work between people who responded in two consecutive pandemic waves (Table 7B).

In order to take an in-depth look on the role of profession in the life of respondents, we used the Utrecht Work Engagement Scale (UWES). One can observe that the well-being and the pleasure of going to work decreased during the last two pandemic waves, compared to first wave, and also that wave 1 is different from waves 2, 4 and 5 in this regard (Table 8A).

**Table 8:** The role of profession in the life of respondents.

A. Different pandemic waves						B. Different consecutive waves (panel samples)					
A.	Wave 1	Wave 2	Wave 3	Wave 4	Wave 5	B.	Wave 1	Wave 2	Wave 3	Wave 4	Wave 5
Wave 1		*0.008*	0.218	*0.010*	*0.002*	Wave 1		0.343	0.414	0.407	0.820
Wave 2			0.061	0.394	0.506	Wave 2			0.800	0.388	0.944
Wave 3				*0.034*	*0.013*	Wave 3				0.813	0.875
Wave 4					0.849	Wave 4					0.962
Wave 5						Wave 5					

(A) Different pandemic waves; (B) different consecutive waves (panel samples-Wilcoxon signed rank test, *p* < 0.05). The italic values are statistically significant (*p*<0.05).

The Mann–Whitney U test also shows statistically significant differences in terms of work engagement. Work commitment increases with positive perception of one’s health, work safety, and the satisfaction of basic psychological needs, as well as with the efficiency of medical protocols. Thus, the raising numbers of successfully treated patients fostered positive and optimistic attitudes towards dealing with the pandemic. Also, diminishing fear and anxiety associated with possible illness/infection with SARS-CoV-2, brought about consistent increases in work engagement; a trend which is observable from one pandemic wave to another.

The Wilcoxon signed rank test indicates no statistically significant differences in terms of scores related to work commitment for the same person from one pandemic wave to another (Table 8B).

During the COVID-19 pandemic, work had a special significance in the lives of the respondents, as shown by the positive correlation between work significance and work commitment, and between the former and the satisfaction of basic psychological needs. Most health workers feel full of energy at work (52%), proud of the work they do (59.5%) and involved in their daily activities (65.5%).

The Mann–Whitney U test shows statistically significant differences between the first and last pandemic waves, and between waves 3 and 5 (Table 9A). The perception of work significance increased towards the end of the pandemic (in wave 5) compared to waves 1 and 3, possibly in the optimistic context created by increasing healing rates.

**Table 9:** The perception of work significance.

A. Different pandemic waves						B. Different consecutive waves (panel samples)					
A.	Wave 1	Wave 2	Wave 3	Wave 4	Wave 5	B.	Wave 1	Wave 2	Wave 3	Wave 4	Wave 5
Wave 1		0.522	0.998	0.213	*0.026*	Wave 1		0.464	0.432	0.585	0.850
Wave 2			0.553	0.522	0.169	Wave 2			0.866	0.700	0.925
Wave 3				0.131	*0.014*	Wave 3				0.058	0.423
Wave 4					0.628	Wave 4					0.345
Wave 5						Wave 5					

(A) Different pandemic waves; (B) different consecutive waves (panel samples used the Wilcoxon signed rank test, *p* < 0.05). The italic values are statistically significant (*p*<0.05).

The average values of recorded scores (wave 1 = 42.91, wave 2 = 43.53, wave 3 = 42.96, wave 4 = 42.03, wave 5 = 40.86) reflect the degree to which people appreciate their work effort makes a positive contribution and brings benefits to others, or to the whole society.

When it comes to work meaningfulness, the Wilcoxon signed rank test does not show statistically significant differences between the answers given by the same people in different pandemic waves. Thus, the meaning of work remains relatively constant for the same person, at least in 2 consecutive waves (Table 9B).

The respondents in our study consider the work carried out during the pandemic as meaningful, with a positive impact on those around them (69.6% of all respondents), which gives them strength and inspire them to deal with difficult situations. A career in this field of work and the professional satisfaction of healing patients significantly contributes to the fulfilment of a meaningful personal life (e.g., “I found a profession whose purpose brings me satisfaction,” 66% answering with a maximum score).

The Mann–Whitney U test emphasises significant differences (*p* < 0.05) regarding the item of satisfying basic psychological needs between wave 1 and waves 2, 3 and 5; and waves 3 and 5 (Table 10A). This means that basic psychological needs were fulfilled towards the end of the pandemic, rather than in the initial (wave 1) or middle (wave 3) stages. On the contrary, the Wilcoxon signed rank test does not show statistically significant differences in the satisfaction of basic psychological needs between responses provided by the same people in successive pandemic waves (Table 10B).

**Table 10:** Perception of satisfying basic psychological needs.

A. Different pandemic waves						B. Different consecutive waves (panel samples)					
A.	Wave 1	Wave 2	Wave 3	Wave 4	Wave 5	B.	Wave 1	Wave 2	Wave 3	Wave 4	Wave 5
Wave 1		*0.032*	*0.010*	0.107	*0.000*	Wave 1		0.347	0.393	0.733	0.183
Wave 2			0.979	0.878	0.070	Wave 2			0.272	0.272	0.147
Wave 3				0.822	*0.015*	Wave 3				0.528	0.625
Wave 4					0.156	Wave 4					0.162
Wave 5						Wave 5					

(A) Different pandemic waves; (B) different consecutive waves (panel samples used the Wilcoxon signed rank test, *p* < 0.05). The italic values are statistically significant (*p*<0.05).

Regarding patient care, the Mann–Whitney U test shows statistically significant differences (*p* < 0.05) between waves 1 and 2 compared to waves 4 and 5. Also, the fourth wave stands out when compared to waves 1, 2, 3 and 5 (Table 11A).

**Table 11:** Patient care.

A. Different pandemic waves						B. Different consecutive waves (panel samples)					
A.	Wave 1	Wave 2	Wave 3	Wave 4	Wave 5	B.	Wave 1	Wave 2	Wave 3	Wave 4	Wave 5
Wave 1		0.437	0.772	*0.009*	*0.000*	Wave 1		0.723	0.590	0.179	*0.001*
Wave 2			0.556	*0.017*	*0.000*	Wave 2			0.713	0.943	*0.004*
Wave 3				*0.012*	*0.000*	Wave 3				0.786	0.250
Wave 4					*0.000*	Wave 4					*0.009*
Wave 5						Wave 5					

(A) Different pandemic waves; (B) different consecutive waves (panel samples used the Wilcoxon signed rank test, *p* < 0.05). The italic values are statistically significant (*p*<0.05).

Average scores (wave 1 = 23.43, wave 4 = 22.21, wave 5 = 12.05) show statistically significant differences, indicating that the basic psychological needs were satisfied by adapting to the new pandemic-related working conditions.

The Wilcoxon signed rank test also shows statistically significant differences between wave 5 and waves 1, 2 and 4 (Table 11B). Panel respondents report the same level of competence when caring for patients, across the pandemic waves. However, participants from the panel samples in wave 5 considered that the medical care provided during the last pandemic wave was of a higher level than the one specific to waves 1, 2 and 4, as shown by the following mean values: wave 1 versus wave 5: 23.92 versus 11.78; wave 2 versus wave 5: 23.63 versus 11.45; wave 4 versus wave 5: 23.88 versus 11.11.

The medical personnel appreciated that the medical care they provided during the pandemic was adequate (82% of respondents), although at the cost of one’s own mental (14.2%) and physical exhaustion (17.1%). It should be highlighted that deeply rooted professional convictions, i.e., caring for patients is the central element in the code of professional conduct in the medical field (88), influence job satisfaction and make medical staff focus on the physical, mental, and emotional wellbeing of patients, even when this task becomes risky or even more demanding.

The Stanford Acute Stress Reaction Questionnaire (Stanford Acute Stress Reaction Questionnaire) was introduced into the research design starting with the second wave. Following the application of the Mann–Whitney U test, no statistically significant differences in terms of scores were obtained when assessing the state of stress across pandemic waves (Table 12A), although average scores recorded in wave 3 (wave 3 = 47.32) are much lower than those recorded in wave 2 (wave 2 = 61.96).

**Table 12:** Stress of infection with COVIS-19.

A. Different pandemic waves						B. Different consecutive waves (panel samples)					
A.	Wave 1	Wave 2	Wave 3	Wave 4	Wave 5	B.	Wave 1	Wave 2	Wave 3	Wave 4	Wave 5
Wave 1		0.089	0.191	0.077	0.055	Wave 1		0.813	0.000	0.462	0.813
Wave 2			0.319	0.469	0.568	Wave 2			0.641	0.701	0.570
Wave 3				0.211	0.097	Wave 3				0.724	0.713
Wave 4					0.696	Wave 4					0.794
Wave 5						Wave 5					

(A) Different pandemic waves, (B) different consecutive waves (panel samples used the Wilcoxon signed rank test, *p* < 0.05).

The Wilcoxon signed rank test does not show statistically significant differences between participants’ responses specific to different pandemic waves (Table 12B).

The Maslach Burnout Inventory scale was introduced starting with wave 3. The results of the Mann–Whitney U test point out statistically significant differences (*p* < 0.05) between wave 3 and waves 4, 5 and also between waves 2 and 3 (Table 13A).

**Table 13:** Professional burnout.

A. Different pandemic waves						B. Different consecutive waves (panel samples)					
A.	Wave 1	Wave 2	Wave 3	Wave 4	Wave 5	B.	Wave 1	Wave 2	Wave 3	Wave 4	Wave 5
Wave 1	NA	NA	NA	NA	NA	Wave 1				*0.045*	0.250
Wave 2			*0.001*	0.783	0.940	Wave 2					0.862
Wave 3				*0.009*	*0.001*	Wave 3					
Wave 4					0.709	Wave 4					
Wave 5						Wave 5					

(A) Different pandemic waves, (B) different consecutive waves (panel samples used the Wilcoxon signed rank test, *p* < 0.05). *NA, no answer.

The mean scores recorded during certain pandemic waves (wave 3 = 17.10, wave 4 = 23.67, wave 5 = 22.49) show a stronger increase in burnout cases towards the end of the COVID-19 pandemic. The Wilcoxon signed rank test shows statistically significant differences between waves 3 and 4 (Table 13B), meaning that professional burnout is prevalent in wave 4, compared to wave 3 (wave 4 = 22.36, wave 3 = 15.18).

#### 4.3.4. Structural model testing

**Figure 2:**
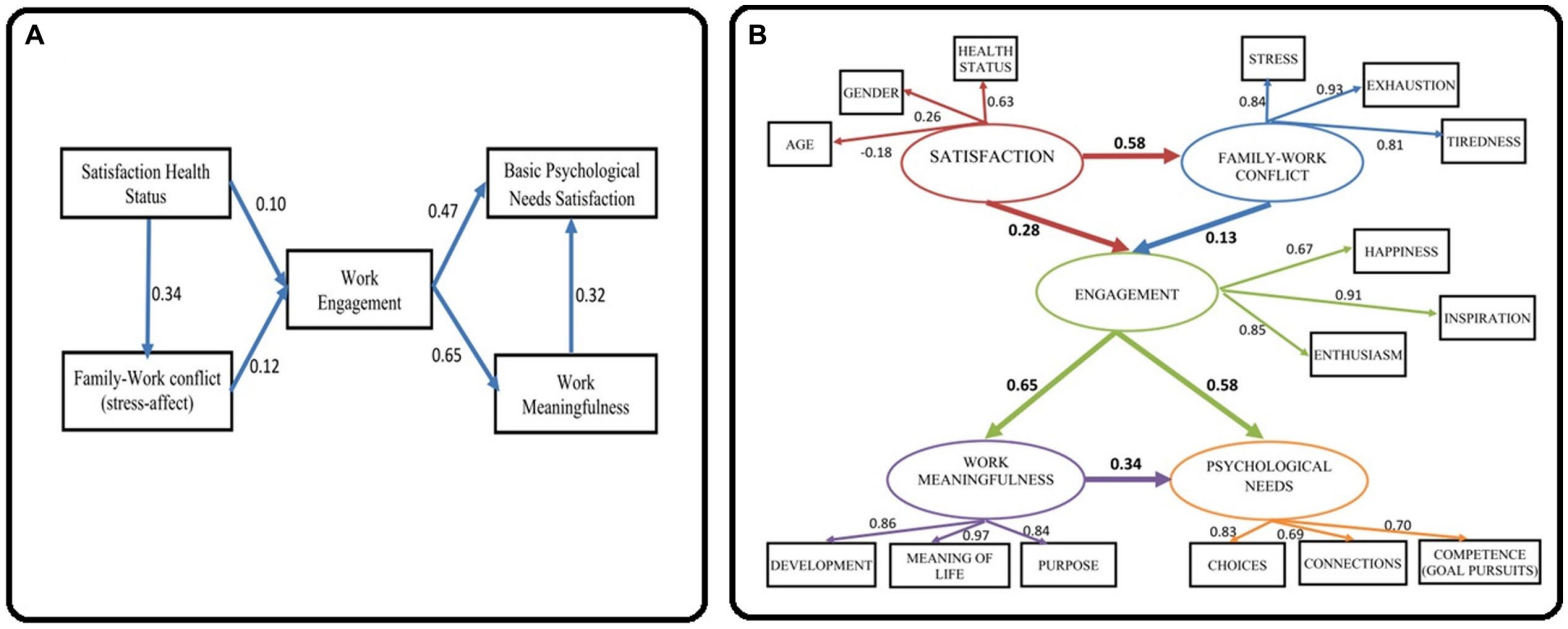
The structural model. **(A)** Hypothesized structural model. **(B)** Graphical representation of the structural model.

The developed model is based on inferential research results from the previous sections. It estimates accommodation mechanisms (causes and effects) to a continuous changing work environment. We hypothesized that the perceived state of health is reflected in the family relations, and in the work involvement. Work engagement gives psychological satisfaction and makes work meaningful which makes one feel fulfilled and valuable (Figure 2A).

Even if results show statistically significant but often week relationships, the parameters for the presented model show a very good-fitting reasonably consistent with the data: SRMR = 0.027, RMSEA = 0.074, CFI = 0.985, TLI = 0.962. All the statistically significant relationships are positive in direction. The level of perceived health as a potential supporting factor in the family–work conflict has a loading factor of 0.34. The relation between perceived heath status and work engagement has a loading factor of 0.10. Well-being at home influences work engagement (loading factor 0.12). Work engagement is an important mediator for the perceived meaningfulness of work (loading factor 0.65) and for satisfying basic psychological needs (loading factor 0.47). Work meaningfulness influences also the satisfaction of basic psychological needs (loading factor 0.32).

Using the structural equation modelling (SEM) procedure we obtained the relationships presented in Figure 2B. The final structural model provides a good fit with all significant paths (SRMR = 0.072, RMSEA = 0.085, CFI = 0.928, TLI = 0.910). In Figure 2B (Graphical representation of the structural model), the measurement model has observed variables shown in rectangles, and latent variables drown as circles; the structural model tests the mediating effects between the latent variables (on the path satisfaction – family–work conflict - work engagement - work meaningfulness - psychological needs); straight lines with an arrow at the end represent the hypothesized effect one variable has on another.

In SEM, the exogenous variable is Satisfaction (mainly the perceived state of health), which has no predictor within the model. All other variables are endogenous or dependent variables (e.g., family–work conflict, work engagement, work meaningfulness, and psychological needs), their values being determined by other variables in the model.

All correlations are positive in direction with one exception: the relation between age and satisfaction level (−0.18). This suggests that youth is a moderate factor of confidentiality regarding perceived health status and satisfaction level, disregarding the sex of participants. The satisfaction level is a predictor of the family-work relation (0.58), both having a moderate direct effect on work engagement.

The family-work relation is saturated by the measured variables exhaustion (“Quite often I come back from work emotionally exhausted, not being able to participate in family life,” 0.93), stress (“The stress from work also affects me at home so that I can no longer do what I like, or makes me happy,” 0.84) and tiredness (“When I get home from work, I am too tired to participate in family activities,” 0.81). The largest direct contribution to work engagement comes from inspiration (“My work inspires me,” 0.91), followed by enthusiasm (“I am enthusiastic about my work,” 0.85), and a general feeling of happiness (“I feel happy when I work hard,” 0.67).

The most consistent effect in the model is that of work engagement on work meaningfulness (0.65). The general disposition that life has meaning presents the highest direct contribution to work meaningfulness (“I understand how my work contributes to the meaning of my life,” 0.97), supporting personal development (“My work contributes to my personal development,” 0.86), and becoming the purpose in life (“My work contributes to a purpose greater than myself,” 0.84). Work engagement moderately satisfies psychological needs (0.34), saturated by the freedom of choice (“At work, I have a sense of choice and freedom in the things I do,” 0.83), competence in achieving goals (“When I am at work, I feel that I am competent to achieve my goals,” 0.70), and social connectivity (“I feel connected to the people who care about me at work and who I care about,” 0.69).

As an important outcome, in SEM there are no correlations with patient care, measuring physicians’ own perceptions of the quality of care they provide to patients, which highlights that, regardless of unfavourable long-term conditions, work involvement provides a high level of health care professionalism.

## 5. Discussion

Although the COVID-19 pandemic was emotionally exhausting, this study shows that the medical staff had a sense of personal achievement due to meaningful work and commitment to work, which is consistent with the results obtained by other research works (89, 90). Respondents in our research advocated an enhance involvement in the activities carried out during the pandemic crisis (65.5%). According to Rana (91), job satisfaction plays an important role in the work commitment and performance of medical professionals. During pandemic conditions, job satisfaction was related to the number of consecutive shifts, occupational well-being, job security, and professional stability at work. The higher the perceived job satisfaction, the higher the job performance and productivity in healthcare (91). In Mukaihata et al. (92) study on psychiatric nurses, work engagement moderated the direct and indirect effects of patient-related stressor on job satisfaction.

Silvia De Simone et al. (93) found correlations between work engagement, job satisfaction, and self-efficacy. During the COVID 19 crisis, research revealed that people with high self-efficacy are more able and comfortable to take on challenging tasks, being more confident in their ability to overcome difficult situations (94); self-efficacy being negatively correlated with anxiety (95, 96).

Our findings highlighted that work engagement and high perceived level of work meaningfulness reduce physicians’ burnout and sustain the quality of patient care. In a systematic review of over 4,700 articles focusing on physicians’ burnout Hodkinson et al. (59) found out that burnout related to low work engagement and meaningfulness, as well as low job satisfaction, and low patient satisfaction.

Similarly, Guerrero-Barona et al. (97) carried out correlations between the quality of family life and work conflict, psychosocial factors, burnout syndrome and emotional intelligence.

Literature concerning work engagement before the COVID 19 crisis indicated that work engagement was positively correlated with the quality of care (24). Other studies examined the association of burnout with the quality of patient care, based on samples from all categories of medical personnel (98, 99). Babenko (100) investigated the role of basic psychological needs (autonomy, competence, and relatedness) in physicians’ professional well-being in terms of job satisfaction, work engagement, and burnout. This study indicated that the need for relatedness had the largest contributions to physicians’ professional life satisfaction, work-related engagement, and exhaustion, respectively.

We consider that these general factors and relationships sharpened their manifestation during pandemic conditions. The uncertainty of the pandemic triggered a mobilization of resources released by physician’s commitment to work. Further, the work engagement raised the awareness of the work meaningfulness under these extreme conditions. Likewise, patient care, becoming the central priority during COVID 19 health crisis conditions, seems to have disconnected from previous influencing factors and relationships.

## 6. Conclusion

The propose of this study was to identify potential affecting factors of healthcare work sustainability during the change-related uncertainty conditions generated by the COVID 19 crisis. Dynamics and relations of nine carefully selected variables and constructs were tracked along all five pandemic waves in Romania, which span from March 2020 to April 2022. The tested variables and constructs are perception of healthcare workers of their own state of health, their workplace safety, the work–family conflict, the satisfaction of basic psychological needs, the work meaningfulness and work engagement, patient care, pandemic stress and burnout.

Key findings can be summarized as follows:

- The analysis identified perceived personal health status as an important factor in the perception of the dangerousness of workplace, the felt pandemic stress, the work–family conflict, the satisfaction of basic psychological needs, the meaning of and commitment to work
- Patient care seems to be above the own health perception and may be associated with the satisfaction of the basic psychological needs of the medical staff, the work-family balance, and the perception of workplace safety
- The sense of belonging (ownership) and work commitment correlate with the quality of patient care and supports the encountered facts that the medical staff managed to find resources to cope with professional stress and burnout during the COVID 19 crisis
- Analysing items dynamics during the 5 pandemic waves, certain adaptive attitudes (e.g., increasing confidence, satisfaction) and behaviors towards COVID 19 pandemic threats emerged related to gained experience and the progress in terms of medical protocols and procedures
- The in-depth structural model identified that the own health status satisfaction is a mediator of the family–work conflict and, together, of the work engagement. In turn, work engagement plays a significant role in satisfying basic psychological needs and supporting work meaningfulness. Work meaningfulness influences also the satisfaction of basic psychological needs.

However, despite these strengths, our study has also some limitations that reduce the generalizability of results. The strongly female-dominated samples, with 74.5% women, although it mirrors the structure of the health personnel in Romania [70.5% female doctors in 2020, according to the National Institute of Statistics (101)] can be a risk of gender bias in the overall generalizability of our findings.

Another drawback is that we have limited data from multiple respondents who are part of all 5 pandemic waves and cannot have a conclusive study in terms of comparing responses between waves. Another limitation of the present research is given by the fact that we did not include in the study a section on the possible infection of medical personnel and the return to work after passing through a COVID 19 disease. Unfortunately, these limitations cannot be improved by further research. Nevertheless, these findings can help medical health systems better identify inner vulnerabilities and strengths, on which coping strategies can be developed to withstand future disturbances.

## Data availability statement

The raw data supporting the conclusions of this article will be made available by the authors, without undue reservation.

## Ethics statement

The studies involving human participants were reviewed and approved by Ethic Committee of Center for Risk Studies Spatial and Dynamic Modeling of Land and Coastal Systems. The patients/participants provided their written informed consent to participate in this study.

## Author contributions

All authors listed have made a substantial, direct, and intellectual contribution to the work, and approved it for publication.

## Conflict of interest

The authors declare that the research was conducted in the absence of any commercial or financial relationships that could be construed as a potential conflict of interest.

## Publisher’s note

All claims expressed in this article are solely those of the authors and do not necessarily represent those of their affiliated organizations, or those of the publisher, the editors and the reviewers. Any product that may be evaluated in this article, or claim that may be made by its manufacturer, is not guaranteed or endorsed by the publisher.
